# CYP450 and drug efflux transporters polymorphism influence clinical outcomes of Thai osimertinib-treated non-small cell lung cancer patients

**DOI:** 10.3389/fphar.2023.1222435

**Published:** 2023-11-06

**Authors:** Teerapat Majam, Chonlaphat Sukasem, Thanyanan Reungwetwattana, Phichai Chansriwong, Chalirmporn Atasilp, Narumol Trachu, Thanaporn Thamrongjirapat, Rattanaporn Sukprasong, Jennis Meanwatthana

**Affiliations:** ^1^ Department of Pharmacy, Faculty of Pharmacy, Mahidol University, Bangkok, Thailand; ^2^ School of Pharmacy, Walailak University, Nakhon Si Thammarat, Thailand; ^3^ Division of Pharmacogenomics and Personalized Medicine, Department of Pathology, Faculty of Medicine Ramathibodi Hospital, Mahidol University, Bangkok, Thailand; ^4^ Laboratory for Pharmacogenomics, Clinical Pathology, Somdetch Phra Debharatana Medical Centre, Ramathibodi Hospital, Bangkok, Thailand; ^5^ Pharmacogenomics and Precision Medicine Clinic, Bumrungrad International Hospital, Bangkok, Thailand; ^6^ Bumrungrad Genomic Medicine Institute (BGMI), Bumrungrad International Hospital, Bangkok, Thailand; ^7^ Division of Medical Oncology, Department of Medicine, Faculty of Medicine Ramathibodi Hospital, Mahidol University, Bangkok, Thailand; ^8^ Chulabhorn International College of Medicine, Thammasat University, Pathum Thani, Thailand; ^9^ Ramathibodi Comprehensive Cancer Center, Faculty of Medicine, Ramathibodi Hospital, Mahidol University, Bangkok, Thailand

**Keywords:** non-small cell lung cancer, pharmacogenetics, single nucleotide polymorphisms (SNPs), osimertinib, drug-metabolizing enzymes, transporters

## Abstract

**Background:** Osimertinib has shown greater efficacy than standard epidermal growth factor receptor tyrosine kinase inhibitors (EGFR-TKIs) and fewer grade 3 or higher adverse drug reactions (ADRs) in patients with advanced non-small cell lung cancer (NSCLC) harboring *epidermal growth factor receptor* (*EGFR*) mutations. However, the clinical outcomes of osimertinib treatment vary depending on the patient’s ethnicity. Therefore, further research is necessary to evaluate the impact of single nucleotide polymorphisms (SNPs) in cytochrome P450 (*CYP450*) and drug transporters on the therapeutic outcomes and ADRs to osimertinib in Thai patients, to provide improved pharmacological treatments for cancer patients.

**Methods:** This retrospective and prospective cohort study enrolled 63 Thai patients with NSCLC treated with 80 mg of osimertinib once daily as monotherapy. Seventeen SNPs in candidate genes related to drug metabolism and transport pathways were analyzed in each patient. Chi-square or Fisher’s exact tests were used to evaluate the associations between SNPs and clinical outcomes, including ADR incidence and objective response rate (ORR). In addition, the correlation between the genotype and median time to treatment failure (TTF) or progression-free survival (PFS) was assessed using Kaplan-Meier analysis and a log-rank test.

**Results:** We identified six SNPs (rs2231142 and rs2622604 in *ABCG2*, rs762551 in *CYP1A2*, rs1057910 in *CYP2C9*, rs28371759 in *CYP3A4*, and *CYP2A6* deletion polymorphism (CYP2A6*4)) that significantly increased the incidence of ADRs. In addition, we found two SNPs (rs2069514 in *CYP1A2* and rs1057910 in *CYP2C9*) that significantly decreased the median TTF, and two SNPs (rs28399433 in *CYP2A6* and rs1057910 in *CYP2C9*) that significantly decreased the median progression-free survival (PFS). Specifically, we found that one of these SNPs (rs1057910 in *CYP2C9*) influenced ADRs, TTF, and PFS. Additionally, SNPs in the *CYP2A6* heterozygous variant (non4/*4) significantly increased ADR incidence, leading to a high frequency of dose reduction (27.0%).

**Conclusion:** Our study demonstrated significant SNPs associated with increased ADR incidence, decreased PFS, and decreased TTF in Thai patients with NSCLC treated with osimertinib. The *CYP2C9* (*3) and *CYP2A6* (*4) allele frequencies differed between ethnicities and were associated with an increased incidence of ADRs. These findings highlight the importance of considering genetic factors in NSCLC treatment and may facilitate personalized medicine approaches. Moreover, our study showed a higher incidence of ADRs than the previous trials, including FLAURA and AURA2, and a higher frequency of dose reduction than reported in the AURA 3 trial, possibly due to genetic differences among the study populations.

## 1 Introduction

Lung cancer is the leading cause of cancer-related deaths, accounting for approximately 18% (Sung et al., 2021). Approximately 80%–85% of these cases are classified as non-small cell lung cancer (NSCLC) (Nicholson et al., 2022). In Thai patients with NSCLC, *epidermal growth factor receptor* (*EGFR*) mutations were most commonly detected, accounting for 68% of cases (Detarkom et al., 2018). Activating *EGFR* mutations have been identified as predictive indicators of sensitivity to first- and second-generation EGFR tyrosine kinase inhibitors (TKIs). Acquired resistance develops 9–12 months after treatment initiation (Westover et al., 2018). One common mechanism underlying acquired resistance is the substitution of threonine with methionine at amino acid position 790 (T790M) in exon 20 of the *EGFR* gene, which accounts for 50%–60% of cases. This mutation impairs the binding of both first- and second-generation EGFR-TKIs by enhancing the ATP-binding affinity of the kinase domain of the EGFR mutant receptor, leading to treatment resistance (Yu et al., 2013).

Osimertinib is a third-generation EGFR-TKI that irreversibly binds to cysteine-797 at the ATP-binding site of the EGFR kinase domain (Li et al., 2023). It potently inhibits *EGFR* phosphorylation in the cases of exon 19 deletion and exon 21 L858R substitution, with IC50 values ranging 13–54 nmol/L. Additionally, in cell lines harboring *EGFR* T790M mutation, osimertinib demonstrates remarkable potency with an IC50 of less than 15 nmol/L. In addition, Osimertinib exhibits high selectivity for mutated EGFR receptors over wild-type EGFR (IC50: 480–1865 nmol/L) (Remon et al., 2018), resulting in less severe gastrointestinal and skin toxicities than those elicited by the first- or second-generation EGFR-TKIs. Additionally, osimertinib has improved overall survival in previously untreated advanced NSCLC patients with *EGFR* mutations compared with standard EGFR-TKIs (Ramalingam et al., 2020). Osimertinib is predominantly metabolized by CYP3A4, CYP2A6, CYP2C9, CYP3A5, and CYP2E1 enzymes, which account for 44.4%, 15.5%, 12.0%, 9.6%, and 3.0% of the metabolism, respectively (Dickinson et al., 2016). It produces at least two circulating metabolites, AZ5104 and AZ7550, accounting for 10% of the parent compound (AstraZeneca, 2021). AZ7550 has a potency and selectivity profile comparable to osimertinib, whereas AZ5104 has an 8-fold greater potency against EGFR mutations (Han et al., 2021).

In a previous study, variability in the steady-state area under the plasma drug concentration-time curve (AUCs) of AZ5104 between ethnic groups was observed, with a 10%–23% decrease in Asian *versus* Caucasian patients; however, the underlying reason remains unclear (Brown et al., 2017). CYP450 predominantly metabolizes Osimertinib and is a substrate for P-glycoprotein (P-gp), which is encoded by *ABCB1,* and breast cancer resistance protein (BCRP), which is encoded by *ABCG2* (AstraZeneca, 2021), which may lead to individual variations in plasma osimertinib concentrations due to genetic polymorphisms. A previous study also found a linear relationship between ADR development and osimertinib concentration, with an increased risk of rash, diarrhea, and QTc prolongation (Brown et al., 2017). Moreover, in Asian patients, the incidence of QTc prolongation was slightly higher than that reported in the FLAURA study (Soria et al., 2018; Cho et al., 2019). Furthermore, a significant association was demonstrated between the AUC_0-24_ of osimertinib, SNPs rs1128503 in *ABCB1*, and SNPs rs2231137 in *ABCG2* with grade 2 or higher adverse events (Ishikawa et al., 2023). The previous studies on EGFR-TKIs found a correlation between SNPs rs762551 in *CYP1A2* and the severity of erlotinib-induced skin rash, along with the development of diarrhea and SNPs rs2470890 in *ABCB1* and rs776746 in *CYP3A5* (Liao et al., 2020). Additionally, SNPs rs2032582 in *ABCB1* were associated with afatinib-induced diarrhea (Sogawa et al., 2020), and reduced function of *CYP2D6* increased the risk of gefitinib-induced rash (Suzumura et al., 2012). However, the association between SNPs in *CYP450* and drug efflux transporters and efficacy outcomes remains unclear. We investigated seventeen SNPs in *CYP450* and drug efflux transporters that may alter the pharmacokinetic and pharmacodynamic profile of osimertinib, intending to advance personalized medicine approaches.

## 2 Materials and methods

### 2.1 Patients and study design

We recruited 63 NSCLC patients for this retrospective and prospective cohort study between June 2022 and January 2023 from the Division of Medical Oncology, Department of Medicine, Faculty of Medicine, Ramathibodi Hospital, Mahidol University, Bangkok, Thailand. The inclusion criteria were histologically confirmed *EGFR* mutation, monotherapy with 80 mg osimertinib once daily, age at diagnosis of >18 years, and normal baseline laboratory findings (complete blood count, renal function test, and liver function test). Patients were excluded if they received other medications that interfered with osimertinib drug levels or if toxicity was reported in the osimertinib-approved product monograph, such as itraconazole, rifampicin, simvastatin, and amiodarone (AstraZeneca, 2021). All patients provided written consent before enrolling in the study, and the study was approved by the Ethics Committee of Ramathibodi (ethics approval code: COA. MURA 2022/370).

### 2.2 Genotyping methods

Genomic DNA was isolated from an EDTA tube (6 mL) using an automatic DNA extraction system (MagNaPure; Roche, Mannheim, Germany). The concentration of DNA was approximately 5 ng/μL, and the A260/A280 ratio was in the range 1.70–2.10. A total of 17 SNPs were genotyped, namely, *ABCB1* rs1128503, *ABCG2* rs1871744, *ABCG2* rs2231142, *ABCG2* rs2231164, *ABCG2* rs2622604, *ABCG2* rs4148157, *CYP1A2* rs1871744, *CYP1A2* rs2069514, *CYP1A2* rs762551, CYP2A6*4, *CYP2A6* rs28399433, *CYP2C9* rs1057910, *CYP2C9* rs1799853, *CYP3A4* rs28371759, *CYP3A5* rs10264272, *CYP3A5* rs776746, and *POR* rs1057868. Genotyping was conducted using real-time PCR ViiA7 (ABI, Foster City, CA, USA), following the manufacturer’s instructions. All samples were analyzed with positive and negative controls in 96-well plates to ensure the authenticity of the results. Genotyping of the candidate genes was performed using a TaqMan real-time PCR assay (ABI, Foster City, CA, USA).

### 2.3 Clinical endpoint assessment

We analyzed the association between the SNPs and clinical outcomes, including the incidence of adverse drug reactions (ADRs), median time to treatment failure (TTF), median progression-free survival (PFS), and objective response rate (ORR). ADRs were periodically assessed by each patient’s physician using the National Cancer Institute Common Terminology Criteria for Adverse Events version 5.0. The Naranjo algorithm was used to determine causality. TTF was the interval between initiating osimertinib treatment and a new locally directed or systemic treatment other than osimertinib monotherapy. PFS was defined as the time from osimertinib treatment initiation to disease progression or death from any cause. ORR was defined as the percentage of study patients who achieved a complete or partial response to treatment within a certain period, as assessed by each patient’s physician according to the Response Evaluation Criteria in Solid Tumors version 1.1.

### 2.4 Statistical analysis

Associations between SNPs and clinical outcomes, including ADRs and ORR, and those between patients’ baseline characteristics and clinical outcomes were evaluated using the appropriate chi-square test or Fisher’s exact test. Genetic polymorphisms were assessed for concordance with Hardy-Weinberg Equilibrium (HWE) using Fisher’s exact test. Linkage disequilibrium was explored using Haploview version 4.0. Univariate and multivariate logistic regression analyses were performed to identify the factors associated with clinical outcomes. The multivariate logistic regression analyses included all variables and all SNPs with a *p*-value of <0.1 from the univariate analysis, which is presented in Supplementary Table S2. The correlation between TTF, PFS, and genotype was assessed using Kaplan-Meier analysis and the log-rank test. Statistical analysis was performed using SPSS version 23 for Windows, and significance was set at *p* < 0.05. The sample size calculation was based on a prospective longitudinal observational cohort study of 53 patients with advanced NSCLC receiving osimertinib therapy, which found a significant association between SNPs rs2231137 in *ABCG2* and grade ≥2 adverse events (*p* = 0.008). Of the *ABCG2* wild-type (G/G) patients, 22 (68.75%) had grade ≥2 adverse events, while all three (100%) of the *ABCG2* mutant-type (A/A) patients had grade ≥2 adverse events (Ishikawa et al., 2023). Therefore, based on the output of sample size calculation from n4Studies for a cohort study with binary outcomes (Ngamjarus, 2016), at least 58 patients were needed.

## 3 Results

### 3.1 Patient characteristics

The patient characteristics are presented in Table 1. Sixty-three patients were included in the study: 20 (31.75%) men and 43 (68.25%) women, with a median age of 68 years (range 60–73). Forty-two patients (66.7%) had *EGFR* exon 19 deletion, nineteen patients (30.2%) had an L858R substitution, one (1.6%) had *EGFR* exon 20 insertion, and one (1.6%) had *EGFR* exon 18 G719X mutation. Forty-six patients (73%) harbored the *EGFR* T790M mutation. Patient characteristics were not significantly associated with the ADRs, TTF, PFS, or ORR. Ten patients (15.9%) received osimertinib as first-line therapy, whereas thirty-six patients (57.1%) and 17 patients (27.0%) received osimertinib as second- and later-line therapy, respectively. The previous systemic therapies included erlotinib (40.8%), gefitinib (35.5%), platinum-doublet chemotherapy (21.9%), and mobocertinib (1.8%). The line of treatment and previous systemic therapy were not significantly associated with the clinical outcomes (*p* > 0.05). All patients were administered 80 mg of osimertinib once daily. At the time of data cutoff, the mean follow-up duration was 18 (10–30) months, and 46 patients (73%) were still receiving osimertinib at the initial dosage. All patients had normal baseline laboratory test results, including those of complete blood count, liver function tests, and renal function tests.

**TABLE 1:** Baseline characteristics of the patients enrolled in the study.

Characteristics	Median [25th – 75th percentile] or number (%)		
	1st line (*n* = 10)	2nd line (*n* = 36)	≥3rd line (*n* = 17)
**Sex**			
Female	8 (80.0)	22 (61.1)	13 (76.5)
Male	2 (20.0)	14 (38.9)	4 (23.5)
**Age (years)**	63.5 [56.0–71.5]	70.5 [61.0–73.0]	66.0 [60.0–72.0]
**BMI (kg/m** ^**2**^ **)**	22.5 [21.2–25.2]	20.8 [19.3–23.8]	21.73 [19.5–23.8]
**ECOG PS**			
0	6 (60.0)	10 (27.8)	6 (35.3)
1	4 (40.0)	16 (44.4)	10 (58.8)
2	0 (0)	9 (25.0)	1 (5.9)
3	0 (0)	1 (2.8)	0 (0)
**Smoking status**			
Never	9 (90.0)	28 (77.8)	14 (82.3)
Former	1 (10.0)	6 (16.7)	1 (5.9)
Current	0 (0)	0 (0)	1 (5.9)
Passive	0 (0)	2 (5.5)	1 (5.9)
**Staging**			
IIIA	0 (0)	1 (2.8)	0 (0)
IV	10 (100.0)	35 (97.2)	17 (100)
**Cerebral metastasis**	2 (20.0)	4 (11.1)	7 (41.2)
**Type of *EGFR* mutation**			
Exon 18 G719x	0 (0)	1 (2.8)	0 (0)
Exon 19 deletion	6 (60.0)	22 (61.1)	14 (82.4)
Exon 20 insertion	0 (0)	1 (2.8)	0 (0)
Exon 21 L858R	4 (40.0)	12 (33.3)	3 (17.6)
Exon 20 T790M	1 (10.0)^a^	29 (80.6)^b^	16 (94.1)^b^
**Dose adjustment**			
Dose holding	1 (10.0)	3 (8.3)	3 (17.6)
Dose reduction	3 (30.0)	9 (25.0)	5 (29.4)

BMI, body mass index; ECOG PS, eastern cooperative oncology group performance status; *EGFR*, epidermal growth factor receptor.

^a^ *De novo EGFR*, Exon 20 T790M mutation.

^b^ Acquired *EGFR*, Exon 20 T790M mutation.

### 3.2 Genotype frequencies

The genotype status of drug-metabolizing enzymes and transporters was determined for all 63 patients (Table 2). No genotype distribution deviated from the Hardy–Weinberg equation.

**TABLE 2:** Genotype and allele frequencies of SNPs were compared between the present study and a previous report (*PharmGKB*, 2021).

SNP-ID	Gene	Genotype	*n*	Identified frequency (%)	Allele	Allele frequency in the present study (%)	Allele frequency in the previous report (%)	HWE *p*-value
rs1128503	*ABCB1*	G/G	9	14.3	G	37.3	38.9	0.69
		G/A	29	46.0	A	62.7	61.1	
		A/A	25	39.7				
rs2231142	*ABCG2*	C/C	31	49.2	C	70.6	70.9	0.99
		C/A	27	42.9	A	29.4	29.1	
		A/A	5	7.9				
rs2231164	*ABCG2*	T/T	18	28.6	T	52.4	50.4	0.75
		T/C	30	47.6	C	47.6	49.6	
		C/C	15	23.8				
rs2622604	*ABCG2*	C/C	38	60.3	C	76.2	81.4	0.18
		C/T	20	31.7	T	23.8	18.6	
		T/T	5	7.9				
rs4148157	*ABCG2*	G/G	38	60.3	G	77.8	75.0	0.49
		G/A	22	34.9	A	22.2	25.0	
		A/A	3	4.8				
rs1871744	*ABCG2*	T/T	27	42.9	T	65.9	72.5	0.15
		T/C	29	46.0	C	34.1	27.5	
		C/C	7	11.1				
rs2069514	*CYP1A2*	G/G	31	49.2	G	70.6	73.0	0.65
		G/A	27	42.9	A	29.4	27.0	
		A/A	5	7.9				
rs762551	*CYP1A2*	C/C	3	4.8	C	31.0	35.4	0.36
		C/A	33	52.4	A	69.0	64.6	
		A/A	27	42.9				

### 3.3 SNPs associated with osimertinib-induced ADRs

Table 3 shows the association between genetic polymorphisms and the overall ADR incidence. We found that SNP rs1057910 in *CYP2C9* was significantly associated with an increased incidence of grade 3 ADRs (*p* = 0.003). Additionally, we identified several SNPs that were significantly associated with an increased incidence of specific ADRs, including rs2622604 in *ABCG2* mutant-type (T/T) and *CYP2A6* heterozygous variant (non*4/*4), which were significantly associated with diarrhea (*p* = 0.011 and *p* = 0.046, respectively). Furthermore, SNPs rs2231142 in *ABCG2* mutant-type (A/A), rs1057910 in *CYP2C9* mutant-type (C/C), rs28371759 in *CYP3A4* heterozygous variant (A/G), and rs762551 in *CYP1A2* wild-type (C/C) were identified. These SNPs were associated with myalgia, grade 3 acneiform rash, QTc prolongation, and bullous dermatitis, respectively (*p* = 0.007, *p* = 0.012, *p* = 0.001, and *p* = 0.006, respectively; Table 4).

**TABLE 3:** Genotype and allele frequencies of SNPs were compared between the present study and a previous report (*PharmGKB*, 2021) (cont.).

SNP-ID	Gene	Genotype	*n*	Identified frequency (%)	Allele	Allele frequency in the present study (%)	Allele frequency in the previous report (%)	HWE *p*-value
CYP2A6*4	*CYP2A6*	non*4/non*4	54	85.7	non*4	92.9	95.3	0.28
		non*4/*4	9	14.3	*4	7.1	4.7	
		*4/*4	0	0				
rs28399433	*CYP2A6*	A/A	43	68.3	A	81.7	86.4	0.19
		A/C	17	27.0	C	18.3	13.6	
		C/C	3	4.8				
rs1799853	*CYP2C9*	C/C	63	100	C	100	99.8	0.95
		C/T	0	0	T	0	0.2	
		T/T	0	0				
rs1057910	*CYP2C9*	A/A	57	90.5	A	94.4	95.6	0.44
		A/C	5	7.9	C	5.6	4.4	
		C/C	1	1.6				
rs28371759	*CYP3A4*	A/A	60	95.2	A	97.6	98.5	0.17
		A/G	3	4.8	G	2.4	1.5	
		G/G	0	0				
rs776746	*CYP3A5*	A/A	5	7.9	A	32.5	27.8	0.27
		A/G	31	49.2	G	67.5	72.2	
		G/G	27	42.9				
rs10264272	*CYP3A5*	G/G	63	100	G	100	100	1.00
		G/A	0	0	A	0	0	
		A/A	0	0				
rs41303343	*CYP3A5*	−/−	63	100	no-insT	100	100	1.00
		-/T	0	0	insT	0	0	
		T/T	0	0				
rs1057868	*POR*	C/C	26	41.3	C	65.1	59.9	0.29
		C/T	30	47.6	T	34.9	40.1	
		T/T	7	11.1				

HWE, Hardy–Weinberg equilibrium; insT, insertion T.

**TABLE 4:** Summary of significant association between SNPs and incidence of specific ADRs.

SNP-ID	Gene	Genotype	Incidence of specific ADRs [*n*/*N* (%)]	Specific ADRs	*p*-Value
rs2231142	*ABCG2*	C/C	0/31 (0)	Myalgia	0.007^a^
		C/A	0/27 (0)		
		A/A	1/5 (20.0)		
rs2622604	*ABCG2*	C/C	7/38 (18.4)	Diarrhea	0.011^a^
		C/T	6/20 (30.0)		
		T/T	4/5 (80.0)		
rs762551	*CYP1A2*	C/C	1/3 (33.3)	Bullous dermatitis	0.006^a^
		C/A	0/33 (0)		
		A/A	0/27 (0)		
CYP2A6^a^4	*CYP2A6*	non^a^4/non^a^4	12/54 (22.2)	Diarrhea	0.046^a^
		non^a^4/^a^4	5/9 (55.6)		
rs1057910	*CYP2C9*	A/A	0/57 (0)	Acneiform rash grade 3	0.012^a^
		A/C	0/5 (0)		
		C/C	1/1 (100)		
rs28371759	*CYP3A4*	A/A	11/60 (18.3)	QTc prolongation (%)	0.001^a^
		A/G	3/3 (100.0)		

*n*, number of ADRs, in the genotype; *N*, number of all cases in the genotype; (%), incidence rate.

^a^ Statistically significant.

### 3.4 SNPs associated with osimertinib efficacy outcomes

After initiating osimertinib, 31 patients (49.2%) had an objective response, including one patient (1.6%) with a complete response and 30 patients (47.6%) with a partial response. In the non-response group, 32 patients (50.8%) did not respond, including 30 patients (47.6%) with stable disease and two patients (3.2%) with progressive disease.

**FIGURE 1:**
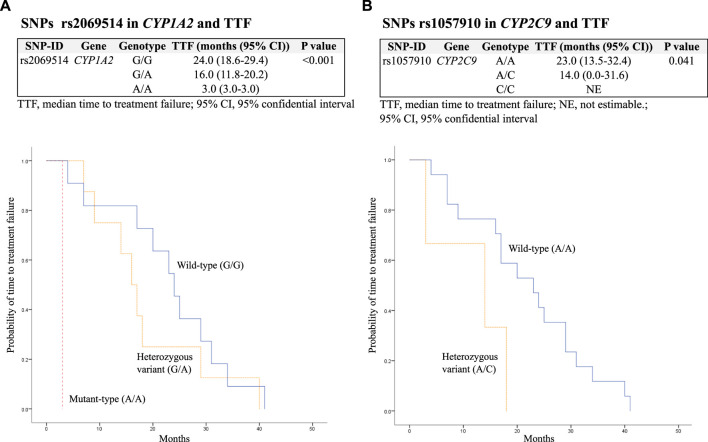
Kaplan–Meier estimates and log-rank tests for median time to treatment failure (TTF) associated with SNPs. **(A)** Association between SNPs rs2069514 in *CYP1A2* and TTF; **(B)** association between SNPs rs1057910 in *CYP2C9* and TTF.

The median TTF was 19 (10.3–29.0) months. In addition, SNPs rs2069514 in *CYP1A2* mutant-type (A/A) and rs1057910 in *CYP2C9* heterozygous variant (A/C) were significantly associated with decreased TTF with *p* < 0.001 and 0.041, respectively. These findings are presented in Table 5 and Figure 1.

**TABLE 5:** Association between SNPs and clinical outcomes.

SNP-ID	Gene	Genotype	All grade ADRs [*n*/*N* (%)]		*p*-Value	Severity of ADRs [*n*/*N* (%)]		*p*-Value	TTF (months (95% CI))	*p*-Value	PFS (months (95% CI))	*p*-Value
			ADRs	Non-ADRs		Grade 1, 2	Grade 3					
rs1128503	*ABCB1*	G/G	9/9 (100)	0/9 (0)	0.150	7/9 (77.8)	2/9 (22.2)	0.682	34.0 (34.0–34.0)	0.216	42.0 (42.0–42.0)	0.283
		G/A	28/29 (96.6)21/25	1/29 (3.4)		25/28 (89.3)	3/28 (10.7)		21.9 (14.3–29.6)		30.3 (24.7–35.8)	
		A/A	(84.0)	4/25 (16.0)		18/21 (85.7)	3/21 (14.3)		16.6 (11.1–22.1)		30.9 (21.7–40.0)	
rs2231142	*ABCG2*	C/C	29/31 (93.5)	2/31 (6.5)	0.557	24/29 (82.8)	5/29 (17.2)	0.607	23.9 (17.4–30.3)	0.420	39.0 (31.4–46.6)	0.081
		C/A	25/27 (92.6)	2/27 (7.4)		22/25 (88.0)	3/25 (12.0)		16.7 (9.0–24.4)		26.7 (20.3–33.2)	
		A/A	4/5 (80.0)	1/5 (20.0)		4/4 (100)	0/4 (0)		21.5 (0.0–46.0)		42.0 (42.0–42.0)	
rs2231164	*ABCG2*	T/T	17/18 (94.4)	1/18 (5.6)	0.669	12/17 (70.6)	5/17 (29.4)	0.055	26.6 (18.3–34.9)	0.128	37.5 (26.7–48.3)	0.287
		T/C	28/30 (93.3)	2/30 (6.7)		25/28 (89.3)	3/28 (10.7)		15.4 (9.2–21.6)		30.0 (22.0–34.0)	
		C/C	13/15 (86.7)	2/15 (13.3)		13/13 (100.0)	0/13 (0)		24.2 (12.8–35.6)		39.0 (31.8–46.2)	
rs2622604	*ABCG2*	C/C	34/38 (89.5)	4/38 (10.5)	0.602	29/34 (85.3)	5/34 (14.7)	0.837	19.6 (13.3–26.0)	0.825	30.0 (24.4–35.6)	0.828
		C/T	19/20 (95.0)	1/20 (5.0)		17/19 (89.5)	2/19 (10.5)		23.3 (10.6–35.9)		42.0 (42.0–42.0)	
		T/T	5/5 (100)	0/5 (0)		4/5 (80.0)	1/5 (20.0)		20.0 (14.1–25.9)		NE	
rs4148157	*ABCG2*	G/G	36/38 (94.7)	2/38 (5.3)	0.447	29/36 (80.6)	7/36 (19.4)	0.271	20.9 (15.4–25.4)	0.930	35.7 (28.3–43.0)	0.529
		G/A	19/22 (86.4)	3/22 (13.6)		18/19 (94.7)	1/19 (5.3)		19.0 (8.3–29.7)		30.5 (25.2–35.9)	
		A/A	3/3 (100)	0/3 (0)		3/3 (100)	0/3 (0)		21.5 (0.0–46.0)		42.0 (42.0–42.0)	
rs1871744	*ABCG2*	T/T	24/27 (88.9)	3/27 (11.1)	0.459	20/24 (83.3)	4/24 (16.7)	0.806	16.8 (9.2–24.4)	0.734	35.7 (28.6–42.8)	0.540
		T/C	28/29 (96.6)	1/29 (3.4)		25/28 (89.3)	3/28 (10.7)		23.3 (15.9–30.6)		29.1 (23.3–34.9)	
		C/C	6/7 (85.7)	1/7 (14.3)		5/6 (83.3)	1/6 (16.7)		27.0 (19.2–34.8)		42.0 (42.0–42.0)	
rs2069514	*CYP1A2*	G/G	28/31 (90.3)	3/31 (9.7)	0.752	23/28 (82.1)	5/28 (17.9)	0.534	24.0 (18.6–29.4)	<0.001^a^	38.4 (33.1–43.7)	0.166
		G/A	25/27 (92.6)	2/27 (7.4)		22/25 (88.0)	3/25 (12.0)		16.0 (11.8–20.2)		26.3 (21.4–31.2)	
		A/A	5/5 (100)	0/5 (0)		5/5 (100)	0/5 (0)		3.0 (3.0–3.0)		22.0 (9.1–34.8)	

*n*, number of ADRs, in the genotype; *N*, number of all cases in the genotype; All grade ADRs, and Severity of ADRs (*N* = 63); TTF, median time to treatment failure (*N* = 20); PFS, median progression-free survival with osimertinib as second-line therapy (*N* = 36); 95% CI, 95% confidence interval; NE, not estimable.

^a^ Statistically significant.

**FIGURE 2:**
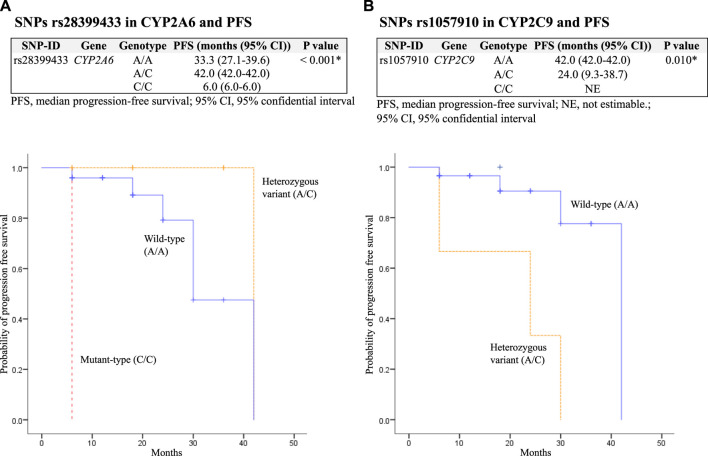
Kaplan–Meier estimates and log-rank tests for median progression-free survival (PFS) associated with SNPs among patients who received osimertinib as second-line therapy. **(A)** association between SNPs rs28399433 in *CYP2A6* and PFS; and **(B)** association between SNPs rs1057910 in *CYP2C9* and PFS.

The median PFS was significantly decreased in patients with SNPs rs28399433 in *CYP2A6* mutant-type (C/C) and rs1057910 in *CYP2C9* heterozygous variant (A/C), with *p* = 0.023 and <0.001, respectively. In addition, among patients who received osimertinib as second-line therapy (*N* = 36), SNPs rs28399433 in *CYP2A6* mutant-type (C/C) and rs1057910 in *CYP2C9* heterozygous variant (A/C) were also significantly associated with decreased PFS, with *p* values of 0.001 and 0.010, respectively (Table 6; Figure 2).

**TABLE 6:** Association between SNPs and clinical outcomes (cont.).

SNP-ID	Gene	Genotype	All grade ADRs [*n*/*N* (%)]		*p*-Value	Severity of ADRs [*n*/*N* (%)]		*p*-Value	TTF (months (95% CI))	*p*-Value	PFS (months (95% CI))	*p*-Value
			ADRs	Non-ADRs		Grade 1, 2	Grade 3					
rs762551	*CYP1A2*	C/C	3/3 (100)	0/3 (0)	0.425	2/3 (66.7)	1/3 (33.3)	0.584	NE	0.987	NE	0.539
		C/A	29/33 (87.9)	4/33 (12.1)		25/29 (86.2)	4/29 (13.8)		20.5 (15.2–25.7)		30.0 (24.6–35.4)	
		A/A	26/27 (96.3)	1/27 (3.7)		23/26 (88.5)	3/26 (11.5)		20.0 (2.2–37.8)		42.0 (42.0–42.0)	
CYP2A6*4	*CYP2A6*	non*4/non*4	49/54 (90.7)	5/54 (9.3)	0.450	42/49 (85.7)	7/49 (14.3)	0.639	21.2 (16.1–26.3)	0.685	34.3 (16.9–52.7)	0.847
		non*4/*4	9/9 (100)	0/9 (0)		8/9 (88.9)	1/9 (11.1)		15.7 (0.0–33.9)		34.8 (28.5–40.1)	
rs28399433	*CYP2A6*	A/A	40/43 (93.0)	3/43 (7.0)	0.721	34/40 (85.0)	6/40 (15.0)	0.767	18.9 (13.1–26.7)	0.181	33.3 (27.1–39.6)	<0.001*
		A/C	15/17 (88.2)	2/17 (11.8)		13/15 (86.7)	2/15 (13.3)		24.3 (15.1–33.6)		42.0 (42.0–42.0)	
		C/C	3/3 (100)	0/3 (0)		3/3 (100)	0/3 (0)		12.0 (0.0–27.7)		6.0 (6.0–6.0)	
rs1057910	*CYP2C9*	A/A	53/57 (93.0)	4/57 (7.0)	0.563	48/53 (90.6)	5/53 (9.4)	0.003*	23.0 (13.5–32.4)	0.041*	42.0 (42.0–42.0)	0.010*
		A/C	4/5 (80.0)	1/5 (20.0)		2/4 (50.0)	2/4 (50.0)		14.0 (0.0–31.6)		24.0 (9.3–38.7)	
		C/C	1/1 (100)	0/1 (0)		0/1 (0)	1/1 (100)		NE		NE	
rs28371759	*CYP3A4*	A/A	55/60 (91.7)	5/60 (8.3)	0.777	48/55 (87.3)	7/55 (12.7)	0.365	20.4 (15.4–25.4)	NE	34.8 (29.8–39.9)	NE
		A/G	3/3 (100)	0/0 (0)		2/3 (66.7)	1/3 (33.3)		NE		NE	
rs776746	*CYP3A5*	A/A	4/5 (80.0)	1/5 (20.0)	0.577	4/4 (100)	0/4 (0)	0.690	27.0 (23.1–30.9)	0.835	30.0 (30.0–30.0)	0.356
		A/G	29/31 (93.5)	2/31 (6.5)		25/29 (86.2)	4/29 (13.8)		22.5 (14.3–30.7)		37.7 (31.7–43.6)	
		G/G	25/27 (92.6)	2/27 (7.4)		21/25 (84.0)	4/25 (16.0)		18.3 (11.1–25.4)		28.6 (21.4–35.9)	
rs1057868	*POR*	C/C	25/26 (96.2)	1/26 (3.8)	0.302	21/25 (84.0)	4/25 (16.0)	0.528	15.8 (6.4–25.2)	0.259	30.0 (19.8–40.2)	0.464
		C/T	26/30 (86.7)	4/30 (13.3)		22/26 (84.6)	4/26 (15.4)		21.9 (16.1–27.7)		42.0 (42.0–42.0)	
		T/T	7/7 (100)	0/7 (0)		7/7 (100)	0/7 (0)		NE		NE	

*n*, number of ADRs, in the genotype; *N*, number of all cases in the genotype; All grade ADRs, and Severity of ADRs (N *=* 63); TTF, median time to treatment failure (*N* = 20); PFS, median progression-free survival with osimertinib as second-line therapy (*N* = 36); 95% CI, 95% confidence interval; NE, not estimable.

^a^ Statistically significant.

### 3.5 Incidence of ADRs

The incidence of ADRs is shown in Table 7. Six patients (9.5%) required a dose hold, and seventeen patients (27.0%) required a dose reduction from the standard prescription owing to ADRs, which included diarrhea in four patients (6.3%), acneiform rash in three patients (4.8%), neutropenia in three patients (4.8%), thrombocytopenia in one patient (1.6%), bullous dermatitis in one patient (1.6%), myositis in one patient (1.6%), transaminitis in one patient (1.6%), QTc prolongation in one patient (1.6%), mucositis in one patient (1.6%), and alopecia in one patient (1.6%). Dose reduction included reducing the dose to 80 mg every other day in 10 patients (15.9%), 80 mg three times a week in 5 patients (7.9%), 80 mg five times a week in 1 patient (1.6%), and 40 mg once daily in 1 patient (1.6%).

**TABLE 7:** Incidence of adverse drug reactions.

Adverse drug reactions	All grades (%)	Grade 1 (%)	Grade 2 (%)	Grade 3 (%)
Acneiform rash	29 (46.0)	15 (23.8)	13 (20.6)	1 (1.6)
Dry skin	23 (36.5)	20 (31.7)	3 (4.8)	0 (0)
Diarrhea	17 (27.0)	11 (17.4)	3 (4.8)	3 (4.8)
QTc prolongation	14 (22.2)	7 (11.1)	3 (4.8)	4 (6.3)
Dry eye	12 (19.0)	11 (17.5)	1 (1.6)	0 (0)
Paronychia	8 (12.7)	6 (9.5)	2 (3.2)	0 (0)
Thrombocytopenia	8 (12.7)	7 (11.1)	1 (1.6)	0 (0)
Mucositis	7 (11.1)	5 (7.9)	2 (3.2)	0 (0)
Transaminitis	5 (7.9)	4 (6.3)	1 (1.6)	0 (0)
Anemia	5 (7.9)	4 (6.3)	1 (1.6)	0 (0)
Neutropenia	4 (6.3)	0 (0)	4 (6.3)	0 (0)
Urticaria	2 (3.2)	1 (1.6)	1 (1.6)	0 (0)
Alopecia	1 (1.6)	0 (0)	1 (1.6)	0 (0)
Bullous dermatitis	1 (1.6)	0 (0)	1 (1.6)	0 (0)
Papulopustular rash	1 (1.6)	0 (0)	0 (0)	1 (1.6)
Paroxysmal AF	1 (1.6)	0 (0)	1 (1.6)	0 (0)
Nausea/vomiting	1 (1.6)	1 (1.6)	0 (0)	0 (0)
Anorexia	1 (1.6)	1 (1.6)	0 (0)	0 (0)
Myalgia	1 (1.6)	1 (1.6)	0 (0)	0 (0)
Myositis	1 (1.6)	0 (0)	0 (0)	1 (1.6)

## 4 Discussion

A recent population pharmacokinetic study showed a linear correlation between exposure to osimertinib, measured using the AUCs of the parent compound and two active metabolites (AZ5104 and AZ7550), and the incidence of ADRs (Brown et al., 2017). AZ5104, in particular, demonstrated an 8-fold greater potency against EGFR mutations (Han et al., 2021). While AZ7550 and AZ5104 are present in approximately 10% of the parent compound (Brown et al., 2017), a decrease in AZ5104 AUCs of 10%–23% in Asian patients compared with that of Caucasian patients may influence clinical outcomes. In addition, a study in Asian populations reported a significant association between SNPs rs1128503 in *ABCB1* and rs2231137 in *ABCG2* and osimertinib-induced grade ≥2 adverse events (Ishikawa et al., 2023). However, the association between SNPs and the efficacy of osimertinib remains unclear, and the specific SNPs that influence the pharmacokinetics and clinical effects of osimertinib in NSCLC remain unknown.

This is the first study to analyze a large number of genetic polymorphisms in candidate genes involved in the osimertinib pharmacokinetic pathway to enable the assessment of both efficacy and safety endpoints. All allele frequencies were consistent with the Hardy–Weinberg equilibrium (*PharmGKB*, 2021). In the present study, the frequency of dose reduction (27.0%) was higher than that reported in the AURA 3 trial (16.5%), and the incidence of ADRs was higher than that in the FLAURA, AURA2, and AURA3 trials (Goss et al., 2016; Mok et al., 2017; Soria et al., 2018). This may be because of genetic differences between patient populations; Asian patients who were administered osimertinib in these studies accounted for only 62%, 63%, and 65% of the patients, respectively. The frequency of the *ABCG2* rs2231164 (C) allele, known as a loss-of-function variant, was 23.72% in the South Asian population and only 12.12% in the European population (Whirl-Carrillo et al., 2021). Furthermore, the frequency of the CYP2A6*4 allele, a slower metabolizer, also differed significantly between ethnic groups (Pang et al., 2015), potentially leading to higher plasma concentrations of osimertinib in the Thai population. These genetic differences may have contributed to the observed differences in dose reductions and ADRs between our study and previous clinical trials. Additionally, we found that the ORR in our study was 49.2%, which was lower than that reported in the AURA 3 trial (71%) (Mok et al., 2017). This difference in response rates may be because of the differences in patient populations between the two studies. In the AURA 3 trial, patients received osimertinib as second-line therapy and 96% of cases and later-line therapy in 4%. In contrast, in our study, 57.1% of patients received osimertinib as second-line therapy, and 27.0% received osimertinib as later-line therapy.

In our study, we identified six SNPs that were significantly associated with the incidence of ADRs, namely, rs2231142 in *ABCG2* mutant-type (A/A), rs2622604 in *ABCG2* mutant-type (T/T), *CYP2A6* heterozygous variant (non*4/*4), rs1057910 in *CYP2C9* mutant-type (C/C), rs28371759 in *CYP3A4* heterozygous variant (A/G) and rs762551 in *CYP1A2* wild-type (C/C). These findings are consistent with previous studies reporting significant associations between genetic polymorphisms and ADR risk. For example, rs2231142 in the *ABCG2* mutant-type (A/A) is significantly associated with sunitinib-induced severe thrombocytopenia (Low et al., 2016), whereas rs2622604 in the *ABCG2* mutant-type (T/T) is significantly associated with irinotecan-induced severe myelosuppression (Cha et al., 2009). Similarly, the *CYP2A6* (*4) allele is significantly associated with letrozole-induced ADRs (Desta et al., 2011), whereas the *CYP3A4* *18 (G) allele is significantly associated with tacrolimus-induced ADRs (Bruckmueller et al., 2015). Notably, in contrast to other genes, rs762551 in the *CYP1A2* wild-type (C/C) was significantly related to osimertinib-induced ADRs. This result may be attributable to the higher enzyme activity observed in the presence of an inducer, such as smoking or heavy coffee consumption, which leads to higher enzyme activity in the *CYP1A2* mutant-type (A/A) (Djordjevic et al., 2010; Wang et al., 2012), and a lower incidence of ADRs was observed in this variant. With regard to efficacy outcomes, we identified two SNPs (rs2069514 in *CYP1A2* and rs1057910 in *CYP2C9*) that were significantly associated with the median TTF and two SNPs (rs28399433 in *CYP2A6* and rs1057910 in *CYP2C9*) that were significantly associated with the median PFS. Notably, one of these SNPs (rs1057910 in *CYP2C9*) was significantly associated with ADRs, TTF, and PFS.

The SNPs in *CYP450* and the drug efflux transporters discussed above were significantly associated with ADRs, TTF, and PFS. Because osimertinib is a substrate of CYP450, ABCB1, and ABCG2 (AstraZeneca, 2021), polymorphisms in these genes may affect the distribution and pharmacokinetics of this drug. Therefore, we hypothesized that mutations causing the decreased function of CYP450, ABCB1, and ABCG2 may affect the tissue distribution and accumulation of osimertinib. An *in vivo* study suggested that ABCB1 and ABCG2 are involved in the tissue accumulation of other TKIs (Al-Shammari et al., 2019). As the active metabolite of osimertinib (AZ5104) is more potent than the parent compound against EGFR mutations (Remon et al., 2018) and accounts for approximately 10% of the parent compound (Brown et al., 2017), the accumulation of osimertinib may lead to a significantly increased incidence of ADRs but significantly decreased TTF and PFS. For example, SNP rs1057910 (C), located in the *CYP2C9* gene, typically encodes the amino acid leucine at position 359, and the resulting allele is also known as CYP2C9*3, which is a decreased function variant (VCV000008408.12 - ClinVar - NCBI, 2013). This variant may lead to poor metabolism of osimertinib in patients carrying the CYP2C9*3 allele, which increases the risk of osimertinib-induced ADRs due to higher osimertinib exposure but also decreases survival outcomes due to lower exposure to its active metabolite (AZ5104).

These findings are similar to those of a previous study on the osimertinib exposure-response relationship, which found that the mortality rate was significantly higher in the high osimertinib drug level group (Rodier et al., 2022). Additionally, the probability of developing rashes, diarrhea, or QTc prolongation increased with exposure, and a linear relationship between adverse event development and osimertinib levels was identified (Brown et al., 2017). The mechanism that supports EGFR-TKI-induced ADRs is the inhibition of EGFR1 and EGFR2 (HER2) signaling, leading to a reduction in growth and impaired healing of the epithelium where EGFR is expressed. This subsequently causes alterations in keratinocyte proliferation and differentiation, reduced growth, impaired healing of the intestinal epithelium, and alterations in myocyte growth (Giovannini et al., 2009; Hirsh et al., 2014; Ikebe et al., 2020).

Our study has several limitations. First, the sample size was small, and some clinical data were retrospectively collected from medical records. Nevertheless, no genotype distribution deviated from the Hardy–Weinberg equation (*PharmGKB*, 2021), and the clinical data were confirmed by the patient’s physician. Second, our inability to control for other confounding factors, such as compliance with osimertinib treatment caused by home oral medication but we had oncology pharmacist pill count measures of compliance at every visit, and all patients maintained a 100% compliance rate. Additionally, there were no observed drug interactions that influenced osimertinib drug levels. However, we did not restrict patient coffee consumption, which may have an impact on higher enzyme activity in the SNP rs762551 in *CYP1A2* mutant-type (A/A). This enzyme was inducible by heavy coffee consumption and has been associated with a lower incidence of ADRs (Djordjevic et al., 2010; Wang et al., 2012). Finally, a pharmacokinetic analysis was not included in this study. However, the association between SNPs and clinical outcomes was consistent with that in previous reports.

In conclusion, our study identified significant SNPs associated with increased ADRs incidence, decreased TTF, and decreased PFS in Thai patients with NSCLC treated with osimertinib. The findings can potentially guide treatment decisions and help optimize individualized therapy for patients with NSCLC harboring EGFR mutations. However, more extensive studies with analysis of osimertinib and its active metabolite drug levels must be conducted to confirm these findings.

## Data Availability

The original contributions presented in the study are included in the article/Supplementary Materials, further inquiries can be directed to the corresponding author.

## Abbreviations

ADR, Adverse drug reactions; BCRP, Breast cancer resistance protein; EGFR, Epidermal growth factor receptor; NSCLC, Non-small cell lung cancer; ORR, Objective response rate; PFS, Progression-free survival; SNP, Single nucleotide polymorphisms; TKI, Tyrosine kinase inhibitors; TTF, Time to treatment failure.

## References

[B1] Al-Shammari A. H. Masuo Y. Fujita K. I. Yoshikawa Y. Nakamichi N. Kubota Y. (2019). Influx and efflux transporters contribute to the increased dermal exposure to active metabolite of regorafenib after repeated oral administration in mice. J. Pharm. Sci. 108 (6), 2173–2179. 10.1016/j.xphs.2019.01.018 30685396

[B2] AstraZeneca (2021). TAGRISSO (osimertinib) [Product monograph]. Mississauga, Ontario: AstraZeneca Canada Inc. Available from: https://www.astrazeneca.ca/content/dam/az-ca/downloads/productinformation/tagrisso-product-monograph-en.pdf.

[B3] Brown K. Comisar C. Witjes H. Maringwa J. de Greef R. Vishwanathan K. (2017). Population pharmacokinetics and exposure-response of osimertinib in patients with non-small cell lung cancer. Br. J. Clin. Pharmacol. 83 (6), 1216–1226. 10.1111/bcp.13223 28009438 PMC5427226

[B4] Bruckmueller H. Werk A. N. Renders L. Feldkamp T. Tepel M. Borst C. (2015). Which genetic determinants should be considered for tacrolimus dose optimization in kidney transplantation? A combined analysis of genes affecting the CYP3A locus. Ther. Drug Monit. 37 (3), 288–295. 10.1097/ftd.0000000000000142 25271728

[B5] Cha P. C. Mushiroda T. Zembutsu H. Harada H. Shinoda N. Kawamoto S. (2009). Single nucleotide polymorphism in ABCG2 is associated with irinotecan-induced severe myelosuppression. J. Hum. Genet. 54 (10), 572–580. 10.1038/jhg.2009.80 19696792

[B6] Cho B. C. Chewaskulyong B. Lee K. H. Dechaphunkul A. Sriuranpong V. Imamura F. (2019). Osimertinib versus standard of care EGFR TKI as first-line treatment in patients with EGFRm advanced NSCLC: FLAURA asian subset. J. Thorac. Oncol. 14 (1), 99–106. 10.1016/j.jtho.2018.09.004 30240852

[B7] CV000008408.12 - ClinVar - NCBI (2013). VCV000008408.12 - ClinVar - NCBI. Available from: https://www.ncbi.nlm.nih.gov/clinvar/variation/8408/.

[B8] Desta Z. Kreutz Y. Nguyen A. T. Li L. Skaar T. Kamdem L. K. (2011). Plasma letrozole concentrations in postmenopausal women with breast cancer are associated with CYP2A6 genetic variants, body mass index, and age. Clin. Pharmacol. Ther. 90 (5), 693–700. 10.1038/clpt.2011.174 21975350 PMC3667657

[B9] Detarkom S. Incharoen P. Jinawat A. Trachu N. Kamprerasart K. Prasongsook N. (2018). P3.09-08 tumor heterogeneity and molecular profile of NSCLC in Thai population. J. Thorac. Oncol. 13 (10), S949–S950. 10.1016/j.jtho.2018.08.1777

[B10] Dickinson P. A. Cantarini M. V. Collier J. Frewer P. Martin S. Pickup K. (2016). Metabolic disposition of osimertinib in rats, dogs, and humans: insights into a drug designed to bind covalently to a cysteine residue of epidermal growth factor receptor. Drug Metabolism Dispos. 44 (8), 1201–1212. 10.1124/dmd.115.069203 27226351

[B11] Djordjevic N. Ghotbi R. Jankovic S. Aklillu E. (2010). Induction of CYP1A2 by heavy coffee consumption is associated with the CYP1A2 −163C>A polymorphism. Eur. J. Clin. Pharmacol. 66 (7), 697–703. 10.1007/s00228-010-0823-4 20390257

[B12] Giovannini M. Gregorc V. Belli C. Roca E. Lazzari C. Viganò M. G. (2009). Clinical significance of skin toxicity due to EGFR-targeted therapies. J. Oncol. 2009, 849051–849058. 10.1155/2009/849051 19584908 PMC2699661

[B13] Goss G. Tsai C. M. Shepherd F. A. Bazhenova L. Lee J. S. Chang G. C. (2016). Osimertinib for pretreated EGFR Thr790Met-positive advanced non-small-cell lung cancer (AURA2): a multicentre, open-label, single-arm, phase 2 study. Lancet Oncol. 17 (12), 1643–1652. 10.1016/s1470-2045(16)30508-3 27751847

[B14] Han L. Zhang X. Wang Z. Zhang X. Zhao L. Fu W. (2021). SH-1028, an irreversible third-generation EGFR TKI, overcomes t790m-mediated resistance in non-small cell lung cancer. Front. Pharmacol. 12, 665253. 10.3389/fphar.2021.665253 33986687 PMC8111447

[B15] Hirsh V. Blais N. Burkes R. Verma S. Croitoru K. (2014). Management of diarrhea induced by epidermal growth factor receptor tyrosine kinase inhibitors. Curr. Oncol. 21 (6), 329–336. 10.3747/co.21.2241 25489260 PMC4257116

[B16] Ikebe S. Amiya R. Minami S. Ihara S. Higuchi Y. Komuta K. (2020). Osimertinib-induced cardiac failure with QT prolongation and torsade de pointes in a patient with advanced pulmonary adenocarcinoma. Int. Cancer Conf. J. 10 (1), 68–71. 10.1007/s13691-020-00450-2 33489705 PMC7797397

[B17] Ishikawa E. Yokoyama Y. Chishima H. Kasai H. Kuniyoshi O. Kimura M. (2023). Population pharmacokinetics, Pharmacogenomics, and adverse events of osimertinib and its two active metabolites, AZ5104 and AZ7550, in Japanese patients with advanced non-small cell lung cancer: a prospective observational study. Investig. New Drugs 41 (1), 122–133. 10.1007/s10637-023-01328-9 36637703 PMC10030409

[B18] Li Y. Mao T. Wang J. Zheng H. Hu Z. Cao P. (2023). Toward the next generation EGFR inhibitors: an overview of osimertinib resistance mediated by EGFR mutations in non-small cell lung cancer. Cell Commun. Signal. 21 (1), 71. 10.1186/s12964-023-01082-8 37041601 PMC10088170

[B19] Liao D. Liu Z. Zhang Y. Liu N. Yao D. Cao L. (2020). Polymorphisms of drug-metabolizing enzymes and transporters contribute to the individual variations of erlotinib steady state trough concentration, treatment outcomes, and adverse reactions in epidermal growth factor receptor–mutated non-small cell lung cancer patients. Front. Pharmacol. 11, 664. 10.3389/fphar.2020.00664 32457635 PMC7225310

[B20] Low S. K. Fukunaga K. Takahashi A. Matsuda K. Hongo F. Nakanishi H. (2016). Association study of a functional variant on ABCG2 gene with sunitinib-induced severe adverse drug reaction. PLOS ONE 11 (2), e0148177. 10.1371/journal.pone.0148177 26914831 PMC4767438

[B21] Mok T. S. Wu Y. L. Ahn M. J. Garassino M. C. Kim H. R. Ramalingam S. S. (2017). Osimertinib or platinum–pemetrexed in EGFR t790m–positive lung cancer. N. Engl. J. Med. 376 (7), 629–640. 10.1056/nejmoa1612674 27959700 PMC6762027

[B22] Ngamjarus C. (2016). n4Studies: sample size calculation for an epidemiological study on a smart device | siriraj medical journal. n4Studies: sample size calculation for an epidemiological study on a smart device | siriraj medical journal. Available from: https://he02.tci-thaijo.org/index.php/sirirajmedj/article/view/58342.

[B23] Nicholson A. G. Tsao M. S. Beasley M. B. Borczuk A. C. Brambilla E. Cooper W. A. (2022). The 2021 WHO classification of lung tumors: impact of advances since 2015. J. Thorac. Oncol. 17 (3), 362–387. 10.1016/j.jtho.2021.11.003 34808341

[B24] Pang C. Liu J. H. Xu Y. S. Chen C. Dai P. G. (2015). The allele frequency of CYP2A6*4 in four ethnic groups of China. Exp. Mol. Pathology 98 (3), 546–548. 10.1016/j.yexmp.2015.03.040 25862079

[B25] PharmGKB (2021). PharmGKB. Available from: https://www.pharmgkb.org/.

[B26] Ramalingam S. S. Vansteenkiste J. Planchard D. Cho B. C. Gray J. E. Ohe Y. (2020). Overall survival with osimertinib in untreated, EGFR-mutated advanced NSCLC. N. Engl. J. Med. 382 (1), 41–50. 10.1056/nejmoa1913662 31751012

[B27] Remon J. Steuer C. Ramalingam S. Felip E. (2018). Osimertinib and other third-generation EGFR TKI in EGFR-mutant NSCLC patients. Ann. Oncol. 29, i20–i27. 10.1093/annonc/mdx704 29462255

[B28] Rodier T. Puszkiel A. Cardoso E. Balakirouchenane D. Narjoz C. Arrondeau J. (2022). Exposure–response analysis of osimertinib in patients with advanced non-small-cell lung cancer. Pharmaceutics 14 (9), 1844. 10.3390/pharmaceutics14091844 36145591 PMC9504753

[B29] Sogawa R. Nakashima C. Nakamura T. Takeuchi K. Kimura S. Komiya K. (2020). Association of genetic polymorphisms with afatinib-induced diarrhoea. Vivo 34 (3), 1415–1419. 10.21873/invivo.11922 PMC7279839 32354939

[B30] Soria J. C. Ohe Y. Vansteenkiste J. Reungwetwattana T. Chewaskulyong B. Lee K. H. (2018). Osimertinib in UntreatedEGFR-mutated advanced non–small-cell lung cancer. N. Engl. J. Med. 378 (2), 113–125. 10.1056/nejmoa1713137 29151359

[B31] Sung H. Ferlay J. Siegel R. L. Laversanne M. Soerjomataram I. Jemal A. (2021). Global cancer statistics 2020: GLOBOCAN estimates of incidence and mortality worldwide for 36 cancers in 185 countries. CA A Cancer J. Clin. 71 (3), 209–249. 10.3322/caac.21660 33538338

[B32] Suzumura T. Kimura T. Kudoh S. Umekawa K. Nagata M. Matsuura K. (2012). Reduced CYP2D6 function is associated with gefitinib-induced rash in patients with non-small cell lung cancer. BMC Cancer 12 (1), 568. 10.1186/1471-2407-12-568 23207012 PMC3536666

[B33] Wang L. Hu Z. Deng X. Wang Y. Zhang Z. Cheng Z. N. (2012). Association between CommonCYP1A2Polymorphisms and theophylline metabolism in non-smoking healthy volunteers. Basic & Clin. Pharmacol. Toxicol. 112 (4), 257–263. 10.1111/bcpt.12038 23167834

[B34] Westover D. Zugazagoitia J. Cho B. Lovly C. Paz-Ares L. (2018). Mechanisms of acquired resistance to first- and second-generation EGFR tyrosine kinase inhibitors. Ann. Oncol. 29, i10–i19. 10.1093/annonc/mdx703 29462254 PMC6454547

[B35] Whirl‐Carrillo M. Huddart R. Gong L. Sangkuhl K. Thorn C. F. Whaley R. (2021). An evidence‐based framework for evaluating Pharmacogenomics knowledge for personalized medicine. Clin. Pharmacol. Ther. 110 (3), 563–572. 10.1002/cpt.2350 34216021 PMC8457105

[B36] Yu H. A. Arcila M. E. Rekhtman N. Sima C. S. Zakowski M. F. Pao W. (2013). Analysis of tumor specimens at the time of acquired resistance to EGFR-TKI therapy in 155 patients with EGFR-mutant lung cancers. Clin. Cancer Res. 19 (8), 2240–2247. 10.1158/1078-0432.ccr-12-2246 23470965 PMC3630270

