# Supplementary material for: CYP450 and drug efflux transporters polymorphism influence clinical outcomes of Thai osimertinib-treated non-small cell lung cancer patients
Source: Front Pharmacol. 2023 Nov 6;14:1222435. doi: 10.3389/fphar.2023.1222435 (PMC10657898; doi:10.3389/fphar.2023.1222435)
Supplement: Supplementary file 1 [file DataSheet1.docx]

**Supplementary Data**

**Study subjects**

***EGFR* mutation Non-small cell lung cancer patients.**

The NSCLC patients who had histologically confirmed diagnoses (N=63) were recruited in this retrospective and prospective cohort study between June 2022 and December 2022 from the Division of Cancer, Department of Medicine, Faculty of Medicine, Ramathibodi Hospital, Mahidol University, Thailand.

**Inclusion criteria**

1. Histologically confirmed *EGFR* mutation
2. Treated with osimertinib
3. Age > 18 years
4. ECOG status 0 - 3
5. Total white blood cell count ≥ 3.5 x10^9^ /L
6. Absolute neutrophil ≥ 1.5x10^9^ /L
7. Hemoglobin level ≥ 9 g/dL
8. Platelet count ≥ 100x10^9^ /L
9. AST and ALT ≤ 2.5 UNL
10. Total bilirubin ≤ 2 mg/dL

**Exclusion criteria**

1. Could not be assessed clinical response data.
2. Could not be assessed ADRs data.
3. Could not be assessed baseline laboratory findings included liver function test, renal function test, complete blood count, CRP, and LDH.
4. Could not be assessed compliance
5. Could not be assessed concurrent medication
6. Could not be assessed previously NSCLC treatment history

**Sample size calculation**

The sample size calculation was based on a prospective longitudinal observational cohort study of 53 patients with advanced NSCLC receiving osimertinib therapy, which identified a significant association between SNPs rs2231137 in *ABCG2* and grade ≥ 2 adverse events (P = 0.008) (Ishikawa et al., 2023). Among the *ABCG2* wild-type (G/G) patients, 22 (68.75%) experienced grade ≥ 2 adverse events, while all three (100%) of the *ABCG2* mutant-type (A/A) patients encountered grade ≥ 2 adverse events. The calculation utilized the equation from Ngamjarus, C., & Chongsuvivatwong, V. (2014). *n4Studies: Sample size and power calculations for iOS.* The Royal Golden Jubilee Ph.D. Program - The Thailand Research Fund & Prince of Songkla University.


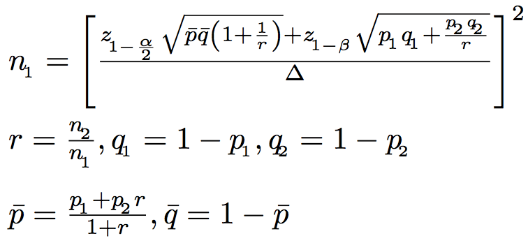


Proportion in group1 (p₁) = 0.6875

Proportion in group2 (p₂) = 1.0

ratio (r) = 22/3 = 7.3

Alpha (α) = 0.05

Beta (β) = 0.20

The sample size was 58 patients

**Clinical data collection**

Patient evaluation at baseline was conducted on the day of osimertinib initiation and included a physical examination and assessment of ECOG status. Patient demographics (age, body weight, height, and gender) and clinical data (osimertinib dose, severity of adverse drug reactions, and any additional medical issues) were obtained from medical records. The clinical outcome evaluation spanned a minimum of 6 months after the initial osimertinib administration. A blood sample was obtained for genotyping assay, using 6 ml EDTA tubes for pharmacogenetic analysis.

**Instruments for DNA extraction**

MagNA Pure Compact automated (Roche Applied Science, Penzberg, Germany)

MagNA Pure Compact tip tray kit (cat. no. 03753166001)

MagNA Pure Compact automated (Roche Applied Science, Penzberg, Germany)

MagNA Pure Compact tip tray kit (cat. no. 03753166001)

MagNA Pure Compact waste tank (cat. no. 03788300001)

MagNA Pure Compact tube rack (cat. no. 03788296001)

MagNA Pure Compact elution tube rack (cat. no. 03788288001)

MagNA Pure Compact drip tray (cat. no. 04347005001)

MagNA Pure Compact cartridge rack (cat. no. 03788237001)

MagNA Pure Compact drop catcher (cat. no. 03788270001)

MagNA Pure Compact nucleic acid isolation kit-large volume (cat. no. 03730972001)

NanoDrop 2000 Spectrophotometer (Thermo Fisher Scientific, DE, USA.)

**Instruments and reagents for Applied Biosystems^®^ ViiA™7 RealTime PCR System**

Applied Biosystems^®^ ViiA™7 Real-Time PCR Instrument

MicroAmp^®^ Fast Optical 96-Well Reaction Plate (0.1mL)

MicroAmp^®^ Optical Adhesive Film

MicroAmp^®^ 96-Well Tray (Black) (0.1µL)

20X, Taqman^®^ Drug Metabolism Genotyping Assay Mix

2X TaqMan^®^ Genotyping Master Mix - Nuclease-free water (PCR grade)

**SNPs genotyping by Taqman RT-PCR**

**Reagents preparation**

The final volume of polymerase chain reaction (PCR) is 20 µL, containing 20 ng (5ng/ µL) of genomic DNA and 10 µL TaqMan SNP genotyping PCR Master Mix, with 1µL of Assay Mix and distilled water 5 µL.

**Amplification**

The condition of PCR was as follows: 95 °C for 10 min to activate the DNA polymerase, followed by 50 cycles of 92 °C for 15 seconds to denature and 60 °C for 90 seconds to anneal and extend.

**Results interpretation**

Analysis was carried out using ViiA™ 7 software (Applied Biosystems). TaqMan Drug Metabolism Genotyping Assays were read at the PCR endpoint. DNA samples on 96-well plates were genotyped simultaneously. Genotype calls for individual samples were made by plotting the normalized intensity of the reporter dyes in each sample well on an allelic discrimination plot. An algorithm in the data analysis software assigns individual sample data to a particular cluster and makes the genotype calls.

**Genotyping Methods**

Peripheral blood was collected in tubes containing ethylenediaminetetraacetic acid (EDTA). Initially, the MagNA Pure Compact System (Roche, Mannheim, Germany) was employed to extract DNA from the blood samples, resulting in a DNA concentration of approximately 5 ng/µL. Subsequently, the purity of the DNA was assessed using nanodrop microvolume technology (Thermo Fisher Scientific, DE, USA). This evaluation was based on the sample's surface tension characteristics when liquified into a column, as determined by the 260/280 ratio. A score within the range of 1.70-2.10 was considered indicative of good purity.

Genotyping was carried out using the TaqMAN real-time PCR ViiA7 instrument (ABI, Foster City, CA, USA), following the manufacturer's instructions. A total of 17 SNPs were studied, including: *ABCB1* rs1128503 (Assay ID: C__7586662_10), *ABCG2* rs2231142 (Assay ID: C__15854163_70), *ABCG2* rs2231164 (Assay ID: C_15922479_10), *ABCG2* rs2622604 (Assay ID: C___9510352_10), *ABCG2* rs4148157 (Assay ID: C__29809590_20), *ABCG2* rs1871744 (Assay ID: C__11945749_10), *CYP1A2* rs2069514 (Assay ID: C__15859191_30), *CYP1A2* rs762551 (Assay ID: C___8881221_40), *CYP2A6**4 (Assay ID: C__33605265_20), *CYP2A6* rs28399433 (Assay ID: C__30634332_10), *CYP2C9* rs1799853 (Assay ID: C__25625805_10), *CYP2C9* rs1057910 (Assay ID: C__27104892_10), *CYP3A4* rs28371759 (Assay ID: C__27859823_20), *CYP3A5* rs776746 (Assay ID: C__26201809_30), *CYP3A5* rs10264272 (Assay ID: C__30203950_10), *CYP3A5* rs41303343 (Assay ID: C__32287188_10), and *POR* rs1057868 (Assay ID: C___8890131_30), the probe primers from Thermo Fisher Scientific, DE, USA.

**Statistical analysis**

Descriptive statistics for patients were presented using means and standard deviations for continuous variables and medians with interquartile ranges for non-normally distributed data. Frequencies and percentages were provided for categorical variables. Fisher's exact test assessed genetic polymorphisms for concordance with Hardy-Weinberg Equilibrium (HWE). Linkage disequilibrium was explored using Haploview version 4.0.

Associations between the SNPs and responses to treatment, adverse drug reactions, and clinical characteristics (such as age, sex, BMI, ECOG status, type of EGFR mutation, tumor stage, cerebral metastasis, smoking status, drug interaction, dose holding, dose reduction, and drug interaction) were examined using either the chi-square test or Fisher's exact test.

Univariate and multivariate logistic regression analyses were conducted to identify factors associated with toxicity and treatment response. All variables and all SNPs with a p-value < 0.1 in the univariate analysis were included in the multivariate logistic regression analyses.

The correlation between TTF, PFS, and genotype was assessed using Kaplan–Meier analysis and a log-rank test. All statistics were calculated using the SPSS version 23 for Windows, and differences were considered to be significant when P-values were < 0.05

**Supplementary Table S1. Genotype data of the patients enrolled in the study.**

| **Patients ID** | **Age** | **Sex** | **Genotype** | | | | | | | | | | | | | | | | |
| --- | --- | --- | --- | --- | --- | --- | --- | --- | --- | --- | --- | --- | --- | --- | --- | --- | --- | --- | --- |
|  |  |  | *ABCB1*  rs1128503 | *ABCG2*  rs2231142 | *ABCG2*  rs2231164 | *ABCG2*  rs2622604 | *ABCG2*  rs4148157 | *ABCG2*  rs1871744 | *CYP1A2*  rs2069514 | *CYP1A2*  rs762551 | *CYP2A6**4 | *CYP2A6*  rs28399433 | *CYP2C9*  rs1799853 | *CYP2C9*  rs1057910 | *CYP3A4*  rs28371759 | *CYP3A5*  rs776746 | *CYP3A5*  rs10264272 | *CYP3A5*  rs41303343 | *POR*  rs1057868 |
| 1 | 75 | Female | G/A | C/A | T/C | C/C (WT) | G/A | T/T (WT) | A/A (MT) | A/A (MT) | non*4/non*4 | A/A (WT) | C/C (WT) | A/A (WT) | A/G | A/A (WT) | G/G (WT) | -/- (WT) | C/T |
| 2 | 73 | Female | A/A (MT) | C/A | C/C (MT) | C/T | G/A | T/C | G/G (WT) | C/A | non*4/non*4 | A/C | C/C (WT) | A/A (WT) | A/A (WT) | A/G | G/G (WT) | -/- (WT) | C/C (WT) |
| 3 | 62 | Male | G/A | C/A | C/C (MT) | C/C (WT) | G/A | T/C | G/A | C/A | non*4/non*4 | A/C | C/C (WT) | A/A (WT) | A/A (WT) | G/G (MT) | G/G (WT) | -/- (WT) | C/T |
| 4 | 71 | Male | A/A (MT) | C/C (WT) | T/C | C/C (WT) | G/G (WT) | T/T (WT) | G/A | C/A | non*4/non*4 | A/C | C/C (WT) | A/A (WT) | A/A (WT) | G/G (MT) | G/G (WT) | -/- (WT) | C/T |
| 5 | 60 | Male | G/A | C/A | T/C | C/C (WT) | G/G (WT) | T/T (WT) | G/A | C/A | non*4/non*4 | A/C | C/C (WT) | A/A (WT) | A/A (WT) | G/G (MT) | G/G (WT) | -/- (WT) | C/T |
| 6 | 59 | Male | A/A (MT) | C/A | C/C (MT) | C/C (WT) | G/A | T/T (WT) | G/A | C/A | non*4/non*4 | A/A (WT) | C/C (WT) | A/C | A/A (WT) | A/G | G/G (WT) | -/- (WT) | C/T |
| 7 | 79 | Female | G/A | C/A | C/C (MT) | C/C (WT) | G/A | T/T (WT) | G/G (WT) | C/A | non*4/non*4 | A/A (WT) | C/C (WT) | A/A (WT) | A/A (WT) | G/G (MT) | G/G (WT) | -/- (WT) | C/C (WT) |
| 8 | 69 | Female | A/A (MT) | A/A (MT) | C/C (MT) | C/C (WT) | G/A | T/T (WT) | G/A | A/A (MT) | non*4/non*4 | A/A (WT) | C/C (WT) | A/A (WT) | A/A (WT) | A/G | G/G (WT) | -/- (WT) | C/T |
| 9 | 75 | Female | G/A | C/C (WT) | C/C (MT) | C/T | G/G (WT) | T/C | G/G (WT) | C/C (WT) | non*4/non*4 | A/A (WT) | C/C (WT) | A/A (WT) | A/A (WT) | G/G (MT) | G/G (WT) | -/- (WT) | C/T |
| 10 | 89 | Female | G/A | C/A | T/C | C/T | G/A | T/C | G/G (WT) | C/A | non*4/*4 | C/C (MT) | C/C (WT) | A/A (WT) | A/A (WT) | G/G (MT) | G/G (WT) | -/- (WT) | C/T |
| 11 | 77 | Female | G/A | C/A | T/C | C/T | G/A | T/C | G/G (WT) | A/A (MT) | non*4/non*4 | A/A (WT) | C/C (WT) | A/A (WT) | A/A (WT) | A/G | G/G (WT) | -/- (WT) | C/C (WT) |
| 12 | 61 | Female | A/A (MT) | C/A | T/C | C/C (WT) | G/A | T/T (WT) | G/G (WT) | A/A (MT) | non*4/non*4 | A/A (WT) | C/C (WT) | A/A (WT) | A/A (WT) | A/A (WT) | G/G (WT) | -/- (WT) | C/T |
| 13 | 57 | Female | G/A | C/C (WT) | T/C | C/C (WT) | G/G (WT) | T/C | G/A | C/A | non*4/*4 | C/C (MT) | C/C (WT) | A/A (WT) | A/A (WT) | A/G | G/G (WT) | -/- (WT) | C/C (WT) |
| 14 | 60 | Male | A/A (MT) | A/A (MT) | C/C (MT) | C/C (WT) | G/A | T/T (WT) | G/G (WT) | A/A (MT) | non*4/non*4 | A/A (WT) | C/C (WT) | A/A (WT) | A/A (WT) | G/G (MT) | G/G (WT) | -/- (WT) | C/T |
| 15 | 66 | Male | G/A | C/C (WT) | T/T (WT) | T/T (MT) | G/G (WT) | C/C (MT) | G/A | A/A (MT) | non*4/non*4 | A/C | C/C (WT) | A/A (WT) | A/A (WT) | A/G | G/G (WT) | -/- (WT) | C/T |
| 16 | 71 | Female | G/A | C/C (WT) | T/C | C/C (WT) | G/G (WT) | T/C | G/G (WT) | C/A | non*4/non*4 | A/A (WT) | C/C (WT) | A/A (WT) | A/A (WT) | A/G | G/G (WT) | -/- (WT) | C/C (WT) |
| 17 | 66 | Female | G/G (WT) | C/C (WT) | T/T (WT) | C/T | G/G (WT) | T/C | G/G (WT) | A/A (MT) | non*4/non*4 | A/C | C/C (WT) | A/A (WT) | A/G | A/G | G/G (WT) | -/- (WT) | T/T (MT) |
| 18 | 58 | Female | G/A | C/C (WT) | T/T (WT) | C/C (WT) | G/G (WT) | T/T (WT) | G/A | C/A | non*4/non*4 | A/A (WT) | C/C (WT) | A/A (WT) | A/A (WT) | A/G | G/G (WT) | -/- (WT) | C/C (WT) |
| 19 | 61 | Female | A/A (MT) | C/A | T/C | C/T | G/A | T/C | G/A | A/A (MT) | non*4/non*4 | A/C | C/C (WT) | A/C | A/G | A/G | G/G (WT) | -/- (WT) | C/C (WT) |
| 20 | 76 | Female | A/A (MT) | C/C (WT) | T/C | C/C (WT) | G/G (WT) | T/C | G/A | A/A (MT) | non*4/non*4 | A/C | C/C (WT) | A/A (WT) | A/A (WT) | A/G | G/G (WT) | -/- (WT) | C/C (WT) |
| 21 | 66 | Female | G/A | C/C (WT) | T/T (WT) | C/C (WT) | G/G (WT) | T/C | G/A | A/A (MT) | non*4/non*4 | A/A (WT) | C/C (WT) | A/A (WT) | A/A (WT) | A/G | G/G (WT) | -/- (WT) | C/C (WT) |
| 22 | 69 | Male | A/A (MT) | C/C (WT) | C/C (MT) | C/C (WT) | G/G (WT) | T/T (WT) | G/A | C/A | non*4/non*4 | A/A (WT) | C/C (WT) | A/A (WT) | A/A (WT) | G/G (MT) | G/G (WT) | -/- (WT) | T/T (MT) |
| 23 | 72 | Female | G/A | C/C (WT) | C/C (MT) | C/C (WT) | G/G (WT) | T/C | G/G (WT) | A/A (MT) | non*4/non*4 | A/A (WT) | C/C (WT) | A/A (WT) | A/A (WT) | A/G | G/G (WT) | -/- (WT) | T/T (MT) |
| v24 | 68 | Male | A/A (MT) | C/C (WT) | T/T (WT) | C/C (WT) | G/G (WT) | T/C | G/G (WT) | C/C (WT) | non*4/non*4 | A/A (WT) | C/C (WT) | A/A (WT) | A/A (WT) | A/G | G/G (WT) | -/- (WT) | C/C (WT) |
| 25 | 71 | Male | G/A | C/C (WT) | C/C (MT) | C/T | G/G (WT) | T/C | G/G (WT) | A/A (MT) | non*4/non*4 | A/A (WT) | C/C (WT) | A/A (WT) | A/A (WT) | G/G (MT) | G/G (WT) | -/- (WT) | C/C (WT) |
| 26 | 85 | Male | A/A (MT) | C/C (WT) | T/C | C/C (WT) | G/A | T/T (WT) | G/A | A/A (MT) | non*4/non*4 | A/A (WT) | C/C (WT) | A/A (WT) | A/A (WT) | A/G | G/G (WT) | -/- (WT) | C/C (WT) |
| 27 | 38 | Female | A/A (MT) | C/A | T/C | C/T | G/G (WT) | T/C | G/G (WT) | C/C (WT) | non*4/non*4 | A/A (WT) | C/C (WT) | A/C | A/A (WT) | G/G (MT) | G/G (WT) | -/- (WT) | C/T |
| 28 | 60 | Female | G/A | C/C (WT) | T/T (WT) | C/C (WT) | G/G (WT) | T/T (WT) | G/G (WT) | C/A | non*4/non*4 | C/C (MT) | C/C (WT) | A/A (WT) | A/A (WT) | G/G (MT) | G/G (WT) | -/- (WT) | C/C (WT) |
| 29 | 68 | Female | G/G (WT) | C/A | T/C | C/C (WT) | G/A | T/T (WT) | G/A | A/A (MT) | non*4/non*4 | A/A (WT) | C/C (WT) | A/A (WT) | A/A (WT) | A/G | G/G (WT) | -/- (WT) | T/T (MT) |
| 30 | 53 | Female | A/A (MT) | C/C (WT) | T/T (WT) | C/T | G/G (WT) | T/C | G/A | C/A | non*4/non*4 | A/A (WT) | C/C (WT) | A/A (WT) | A/A (WT) | A/G | G/G (WT) | -/- (WT) | C/C (WT) |
| 31 | 84 | Male | G/G (WT) | C/A | T/T (WT) | C/C (WT) | G/G (WT) | T/T (WT) | G/G (WT) | C/A | non*4/*4 | A/A (WT) | C/C (WT) | A/A (WT) | A/A (WT) | G/G (MT) | G/G (WT) | -/- (WT) | C/C (WT) |
| 32 | 69 | Female | G/A | C/A | C/C (MT) | C/C (WT) | G/A | T/C | G/G (WT) | C/A | non*4/non*4 | A/A (WT) | C/C (WT) | A/A (WT) | A/A (WT) | G/G (MT) | G/G (WT) | -/- (WT) | C/T |
| 33 | 80 | Female | G/G (WT) | C/C (WT) | T/T (WT) | T/T (MT) | G/G (WT) | C/C (MT) | G/G (WT) | C/A | non*4/non*4 | A/A (WT) | C/C (WT) | A/A (WT) | A/A (WT) | A/G | G/G (WT) | -/- (WT) | C/T |
| 34 | 68 | Female | A/A (MT) | C/A | T/C | C/T | G/G (WT) | T/C | G/A | C/A | non*4/non*4 | A/A (WT) | C/C (WT) | A/A (WT) | A/A (WT) | A/G | G/G (WT) | -/- (WT) | C/T |
| 35 | 59 | Female | A/A (MT) | C/A | T/C | C/C (WT) | G/A | T/C | G/A | A/A (MT) | non*4/non*4 | A/A (WT) | C/C (WT) | A/A (WT) | A/A (WT) | G/G (MT) | G/G (WT) | -/- (WT) | C/C (WT) |
| 36 | 73 | Male | A/A (MT) | C/A | T/C | C/T | G/A | T/C | G/A | C/A | non*4/non*4 | A/C | C/C (WT) | A/A (WT) | A/A (WT) | A/G | G/G (WT) | -/- (WT) | C/C (WT) |
| 37 | 76 | Male | G/A | C/A | T/C | C/C (WT) | G/G (WT) | T/T (WT) | A/A (MT) | A/A (MT) | non*4/non*4 | A/A (WT) | C/C (WT) | A/C | A/A (WT) | G/G (MT) | G/G (WT) | -/- (WT) | C/C (WT) |
| 38 | 72 | Female | A/A (MT) | C/A | T/C | C/C (WT) | G/G (WT) | T/T (WT) | G/A | C/A | non*4/non*4 | A/A (WT) | C/C (WT) | A/A (WT) | A/A (WT) | G/G (MT) | G/G (WT) | -/- (WT) | C/C (WT) |
| 39 | 54 | Female | G/G (WT) | C/C (WT) | T/T (WT) | C/C (WT) | G/G (WT) | T/T (WT) | G/G (WT) | A/A (MT) | non*4/non*4 | A/A (WT) | C/C (WT) | A/A (WT) | A/A (WT) | G/G (MT) | G/G (WT) | -/- (WT) | C/C (WT) |
| 40 | 68 | Female | G/A | C/A | T/C | C/C (WT) | G/A | T/T (WT) | G/A | A/A (MT) | non*4/non*4 | A/A (WT) | C/C (WT) | A/A (WT) | A/A (WT) | G/G (MT) | G/G (WT) | -/- (WT) | C/T |

ID, identification number; WT, wild-type; MT, mutant-type

**Supplementary Table S1. Genotype data of the patients enrolled in the study. (cont.)**

| **Patients ID** | **Age** | **Sex** | **Genotype** | | | | | | | | | | | | | | | | |
| --- | --- | --- | --- | --- | --- | --- | --- | --- | --- | --- | --- | --- | --- | --- | --- | --- | --- | --- | --- |
|  |  |  | *ABCB1*  rs1128503 | *ABCG2*  rs2231142 | *ABCG2*  rs2231164 | *ABCG2*  rs2622604 | *ABCG2*  rs4148157 | *ABCG2*  rs1871744 | *CYP1A2*  rs2069514 | *CYP1A2*  rs762551 | *CYP2A6**4 | *CYP2A6*  rs28399433 | *CYP2C9*  rs1799853 | *CYP2C9*  rs1057910 | *CYP3A4*  rs28371759 | *CYP3A5*  rs776746 | *CYP3A5*  rs10264272 | *CYP3A5*  rs41303343 | *POR*  rs1057868 |
| 41 | 60 | Male | A/A (MT) | C/C (WT) | T/C | C/T | G/G (WT) | C/C (MT) | G/G (WT) | A/A (MT) | non*4/non*4 | A/A (WT) | C/C (WT) | A/A (WT) | A/A (WT) | G/G (MT) | G/G (WT) | -/- (WT) | T/T (MT) |
| 42 | 77 | Female | G/A | C/C (WT) | T/T (WT) | C/T | G/G (WT) | C/C (MT) | G/G (WT) | C/A | non*4/non*4 | A/A (WT) | C/C (WT) | A/A (WT) | A/A (WT) | A/G | G/G (WT) | -/- (WT) | C/C (WT) |
| 43 | 68 | Female | A/A (MT) | C/C (WT) | T/T (WT) | C/T | G/G (WT) | C/C (MT) | G/G (WT) | C/A | non*4/non*4 | A/C | C/C (WT) | A/A (WT) | A/A (WT) | A/G | G/G (WT) | -/- (WT) | C/C (WT) |
| 44 | 61 | Male | G/A | C/C (WT) | T/C | C/T | G/G (WT) | T/C | G/G (WT) | C/A | non*4/non*4 | A/C | C/C (WT) | A/A (WT) | A/A (WT) | A/G | G/G (WT) | -/- (WT) | C/T |
| 45 | 72 | Female | A/A (MT) | C/C (WT) | T/T (WT) | C/C (WT) | G/G (WT) | T/C | G/A | C/A | non*4/non*4 | A/A (WT) | C/C (WT) | A/C | A/A (WT) | A/G | G/G (WT) | -/- (WT) | C/T |
| 46 | 73 | Male | G/A | C/A | T/C | C/C (WT) | G/A | T/C | G/G (WT) | C/A | non*4/non*4 | A/A (WT) | C/C (WT) | A/A (WT) | A/A (WT) | A/A (WT) | G/G (WT) | -/- (WT) | C/T |
| 48 | 52 | Male | G/A | A/A (MT) | C/C (MT) | C/C (WT) | A/A (MT) | T/T (WT) | G/A | C/A | non*4/*4 | A/A (WT) | C/C (WT) | A/A (WT) | A/A (WT) | A/G | G/G (WT) | -/- (WT) | C/C (WT) |
| 49 | 32 | Male | G/A | C/C (WT) | T/T (WT) | C/C (WT) | G/G (WT) | T/T (WT) | G/G (WT) | C/A | non*4/*4 | A/A (WT) | C/C (WT) | C/C (MT) | A/A (WT) | A/G | G/G (WT) | -/- (WT) | C/T |
| 51 | 72 | Male | G/A | C/C (WT) | T/T (WT) | C/C (WT) | G/G (WT) | T/T (WT) | G/G (WT) | C/A | non*4/non*4 | A/C | C/C (WT) | A/A (WT) | A/A (WT) | G/G (MT) | G/G (WT) | -/- (WT) | C/T |
| 53 | 57 | Female | G/A | C/A | T/C | C/C (WT) | G/A | T/T (WT) | G/A | C/A | non*4/non*4 | A/C | C/C (WT) | A/A (WT) | A/A (WT) | A/A (WT) | G/G (WT) | -/- (WT) | C/T |
| 54 | 71 | Female | G/G (WT) | C/C (WT) | T/T (WT) | T/T (MT) | G/G (WT) | C/C (MT) | A/A (MT) | A/A (MT) | non*4/*4 | A/A (WT) | C/C (WT) | A/A (WT) | A/A (WT) | G/G (MT) | G/G (WT) | -/- (WT) | C/T |
| 55 | 60 | Female | A/A (MT) | C/C (WT) | T/T (WT) | T/T (MT) | G/G (WT) | C/C (MT) | G/G (WT) | A/A (MT) | non*4/non*4 | A/C | C/C (WT) | A/A (WT) | A/A (WT) | G/G (MT) | G/G (WT) | -/- (WT) | C/T |
| 58 | 53 | Female | A/A (MT) | C/A | T/C | C/T | G/G (WT) | T/C | G/A | A/A (MT) | non*4/non*4 | A/A (WT) | C/C (WT) | A/A (WT) | A/A (WT) | A/G | G/G (WT) | -/- (WT) | C/C (WT) |
| 59 | 75 | Female | G/A | C/C (WT) | T/C | C/T | G/G (WT) | T/C | G/A | C/A | non*4/non*4 | A/A (WT) | C/C (WT) | A/A (WT) | A/A (WT) | A/A (WT) | G/G (WT) | -/- (WT) | C/T |
| 60 | 67 | Female | G/A | C/A | T/C | C/T | G/G (WT) | T/C | G/A | C/A | non*4/*4 | A/A (WT) | C/C (WT) | A/A (WT) | A/A (WT) | G/G (MT) | G/G (WT) | -/- (WT) | C/T |
| 61 | 70 | Female | G/G (WT) | A/A (MT) | C/C (MT) | C/C (WT) | A/A (MT) | T/T (WT) | G/G (WT) | A/A (MT) | non*4/*4 | A/A (WT) | C/C (WT) | A/A (WT) | A/A (WT) | A/G | G/G (WT) | -/- (WT) | C/T |
| 62 | 52 | Female | A/A (MT) | C/C (WT) | T/C | C/T | G/G (WT) | T/C | G/A | A/A (MT) | non*4/non*4 | A/A (WT) | C/C (WT) | A/A (WT) | A/A (WT) | A/G | G/G (WT) | -/- (WT) | C/C (WT) |
| 63 | 61 | Female | A/A (MT) | C/A | T/C | C/C (WT) | G/A | T/T (WT) | G/G (WT) | C/A | non*4/non*4 | A/C | C/C (WT) | A/A (WT) | A/A (WT) | G/G (MT) | G/G (WT) | -/- (WT) | C/T |
| 64 | 71 | Female | A/A (MT) | C/C (WT) | T/C | T/T (MT) | G/G (WT) | T/C | G/G (WT) | C/A | non*4/non*4 | A/A (WT) | C/C (WT) | A/A (WT) | A/A (WT) | G/G (MT) | G/G (WT) | -/- (WT) | C/T |
| 73 | 60 | Female | G/A | C/A | C/C (MT) | C/C (WT) | G/A | T/T (WT) | A/A (MT) | A/A (MT) | non*4/*4 | A/A (WT) | C/C (WT) | A/A (WT) | A/A (WT) | A/G | G/G (WT) | -/- (WT) | T/T (MT) |
| 79 | 73 | Female | G/G (WT) | A/A (MT) | C/C (MT) | C/C (WT) | A/A (MT) | T/T (WT) | G/G (WT) | A/A (MT) | non*4/non*4 | A/A (WT) | C/C (WT) | A/A (WT) | A/A (WT) | A/G | G/G (WT) | -/- (WT) | C/T |
| 81 | 70 | Female | G/A | C/C (WT) | T/T (WT) | C/C (WT) | G/G (WT) | T/T (WT) | A/A (MT) | A/A (MT) | non*4/non*4 | A/C | C/C (WT) | A/A (WT) | A/A (WT) | G/G (MT) | G/G (WT) | -/- (WT) | C/C (WT) |
| 83 | 73 | Male | G/G (WT) | C/A | T/C | C/T | G/A | T/C | G/G (WT) | C/A | non*4/non*4 | A/C | C/C (WT) | A/A (WT) | A/A (WT) | G/G (MT) | G/G (WT) | -/- (WT) | T/T (MT) |

ID, identification number; WT, wild-type; MT, mutant-type

**Supplementary Table S2. Association between genetic polymorphisms and incidence of ADRs (univariate analysis). [n/N (%)]**

| **SNP-ID** | **Gene** | **Genotype** | **Dry skin (%)** | **P value** | **Alopecia (%)** | **P value** | **Acne (%)** | **P value** | **Urticaria (%)** | **P value** | **Bullous (%)** | **P value** | **Papule (%)** | **P value** | **Paronychia (%)** | **P value** | **Diarrhea (%)** | **P value** | **QTc (%)** | **P value** | **Myalgia (%)** | **P value** |
| --- | --- | --- | --- | --- | --- | --- | --- | --- | --- | --- | --- | --- | --- | --- | --- | --- | --- | --- | --- | --- | --- | --- |
| rs2231142 | *ABCG2* | C/C  C/A  A/A | 10/31 (32.3)  12/27 (44.4)  1/5 (20.0) | 0.458 | 0/31 (0)  1/27 (3.7)  0/5 (0) | 0.058 | 1/31 (3.2)  0/27 (0)  0/5 (0) | 0.751 | 0/31 (0)  0/27 (0)  2/5 (40.0) | <0.001* | 1/31 (3.2)  0/27 (0)  0/5 (0) | 0.592 | 1/31 (3.2)  0/27 (0)  0/5 (0) | 0.592 | 4/31 (12.9)  4/27 (14.8)  0/5 (0) | 0.658 | 9/31 (29.0)  6/27 (22.2)  2/5 (40.0) | 0.633 | 7/31 (22.6)  7/27 (25.9)  0/5 (0) | 0.776 | 0/31 (0)  0/27 (0)  1/5 (20.0) | 0.003* |
| rs1871744 | *ABCG2* | T/T  T/C  C/C | 9/27 (33.3)  11/29 (37.9)  3/7 (42.9) | 0.876 | 1/27 (3.7)  0/29 (0)  0/7 (0) | 0.508 | 1/27 (3.7)  0/29 (0)  0/7 (0) | 0.847 | 2/27 (7.4)  0/29 (0)  0/7 (0) | 0.252 | 0/27 (0)  1/29 (3.4)  0/7 (0) | 0.551 | 0/27 (0)  0/29 (0)  1/7 (14.3) | 0.017* | 1/27 (3.7)  4/29 (13.8)  3/7 (42.9) | 0.021* | 6/27 (22.2)  8/29 (27.6)  3/7 (42.9) | 0.704 | 4/27 (14.8)  7/29 (24.1)  3/7 (42.9) | 0.600 | 1/27 (3.7)  0/29 (0)  0/7 (0) | 0.508 |
| rs2231164 | *ABCG2* | T/T  T/C  C/C | 4/18 (22.2)  14/30 (46.7)  5/15 (33.3) | 0.225 | 0/18 (0)  1/30 (3.3)  0/15 (0) | 0.572 | 1/18 (5.6)  0/30 (0)  0/15 (0) | 0.255 | 0/18 (0)  0/30 (0)  2/15 (13.3) | 0.037* | 0/18 (0)  0/30 (0)  1/15 (6.7) | 0.197 | 1/18 (5.6)  0/30 (0)  0/15 (0) | 0.281 | 3/18 (16.7)  4/30 (13.3)  1/15 (6.7) | 0.684 | 6/18 (33.3)  7/30 (23.3)  4/15 (26.7) | 0.750 | 6/18 (33.3)  7/30 (23.3)  1/15 (6.7) | 0.244 | 0/18 (0)  0/30 (0)  1/15 (6.7) | 0.197 |
| rs2622604 | *ABCG2* | C/C  C/T  T/T | 13/38 (34.2)  9/20 (45.0)  1/5 (20.0) | 0.523 | 1/38 (2.6)  0/20 (0)  0/5 (0) | 0.716 | 1/38 (2.6)  0/20 (0)  0/5 (0) | 0.947 | 2/38 (5.3)  0/20 (0)  0/5 (0) | 0.507 | 0/38 (0)  1/20 (5.0)  0/5 (0) | 0.335 | 0/38 (0)  0/20 (0)  1/5 (20.0) | 0.003* | 2/38 (5.3)  4/20 (20.0)  2/5 (40.0) | 0.045* | 7/38 (18.4)  6/20 (30.0)  4/5 (80.0) | 0.048* | 6/38 (15.8)  6/20 (30.0)  2/5 (40.0) | 0.558 | 1/38 (2.6)  0/20 (0)  0/5 (0) | 0.716 |
| rs4148157 | *ABCG2* | G/G  G/A  A/A | 13/38 (34.2)  9/22 (40.9)  1/3 (33.3) | 0.868 | 0/38 (0)  1/22 (4.5)  0/3 (0) | 0.388 | 1/38 (2.6)  0/22 (0)  0/3 (0) | 0.893 | 0/38 (0)  1/22 (4.5)  1/3 (33.3) | 0.006* | 1/38 (2.6)  0/22 (0)  0/3 (0) | 0.716 | 1/38 (2.6)  0/22 (0)  0/3 (0) | 0.716 | 6/38 (15.8)  2/22 (9.1)  0/3 (0) | 0.600 | 10/38 (26.3)  6/22 (27.3)  1/3 (33.3) | 0.984 | 9/38 (23.7)  5/22 (22.7)  0/3 (0) | 0.586 | 0/38 (0)  1/22 (4.5)  0/3 (0) | 0.388 |
| rs2069514 | *CYP1A2* | G/G  G/A  A/A | 12/31 (38.7)  9/27 (33.3)  2/5 (40.0) | 0.901 | 0/31 (0)  0/27 (0)  1/5 (20.0) | 0.003* | 1/31 (3.2)  0/27 (0)  0/5 (0) | 0.531 | 0/31 (0)  2/27 (7.4)  0/5 (0) | 0.252 | 1/31 (3.2)  0/27 (0)  0/5 (0) | 0.592 | 1/31 (3.2)  0/27 (0)  0/5 (0) | 0.592 | 4/31 (12.9)  3/27 (11.1)  1/5 (20.0) | 0.859 | 7/31 (22.6)  8/27 (29.6)  2/5 (40.0) | 0.765 | 7/31 (22.6)  5/27 (18.5)  2/5 (40.0) | 0.395 | 0/31 (30)  1/27 (3.7)  0/5 (0) | 0.508 |
| rs762551 | *CYP1A2* | C/C  C/A  A/A | 0/3 (0)  15/33 (45.5)  8/27 (29.6) | 0.181 | 0/3 (0)  0/33 (0)  1/27 (3.7) | 0.508 | 0/3 (0)  1/33 (3.0)  0/27 (0) | 0.888 | 0/3 (0)  1/33 (3.0)  1/27 (3.7) | 0.939 | 1/3 (33.3)  0/33 (0)  0/27 (0) | <0.001* | 0/3 (0)  0/33 (0)  1/27 (3.7) | 0.508 | 1/3 (33.3)  3/33 (9.1)  4/27 (14.8) | 0.439 | 0/3 (0)  10/33 (30.3)  7/27 (25.9) | 0.844 | 1/3 (33.3)  5/33 (15.2)  8/27 (29.6) | 0.051 | 0/3 (0)  0/33 (0)  1/27 (3.7) | 0.508 |
| CYP2A6*4 | *CYP2A6* | non*4/non*4  non*4/*4 | 18/54 (33.3)  5/9 (55.6) | 0.181 | 1/54 (1.9)  0/9 (0) | 0.857 | 0/54 (0)  1/9 (11.1) | 0.043* | 1/54 (1.9)  1/9 (11.1) | 0.267 | 1/54 (1.9)  0/9 (0) | 0.857 | 1/54 (1.9)  0/9 (0) | 0.857 | 6/54 (11.1)  2/9 (22.2) | 0.320 | 12/54 (22.2)  5/9 (55.6) | 0.031* | 13/54 (24.1)  1/9 (11.1) | 0.409 | 1/54 (1.9)  0/9 (0) | 0.857 |
| rs28399433 | *CYP2A6* | A/A  A/C  C/C | 17/43 (39.5)  3/17 (17.6)  3/3 (100.0) | 0.018* | 1/43 (2.3)  0/17 (0)  0/3 (0) | 0.790 | 1/43 (2.3)  0/17 (0)  0/3 (0) | 0.540 | 2/43 (4.7)  0/17 (0)  0/3 (0) | 0.619 | 1/43 (2.3)  0/17 (0)  0/3 (0) | 0.790 | 0/43 (0)  1/17 (5.9)  0/3 (0) | 0.253 | 6/43 (14.0)  2/17 (11.8)  0/3 (0) | 0.775 | 11/43 (25.6)  5/17 (29.4)  1/3 (33.3) | 0.979 | 8/43 (18.6)  5/17 (29.4)  1/3 (33.3) | 0.279 | 1/43 (2.3)  0/17 (0)  0/3 (0) | 0.790 |
| rs1057910 | *CYP2C9* | A/A  A/C  C/C | 22/57 (38.6)  0/5 (0)  1/1 (100) | 0.094 | 1/57 (1.8)  0/5 (0)  0/1 (0) | 0.948 | 0/57 (0)  0/5 (0)  1/1 (100) | <0.001* | 2/57 (3.5)  0/5 (0)  0/1 (0) | 0.897 | 1/57 (1.8)  0/5 (0)  0/1 (0) | 0.948 | 1/57 (1.8)  0/5 (0)  0/1 (0) | 0.948 | 8/57 (14.0)  0/5 (0)  0/1 (0) | 0.617 | 14/57 (24.6)  2/5 (40.0)  1/1 (100) | 0.176 | 11/57 (19.3)  3/5 (60.0)  0/1 (0) | 0.308 | 1/57 (1.8)  0/5 (0)  0/1 (0) | 0.948 |
| rs28371759 | *CYP3A4* | A/A  A/G | 22/60 (36.7)  1/3 (33.3) | 0.701 | 0/60 (0)  1/3 (33.3) | 0.048* | 1/60 (1.7)  0/3 (0) | 0.891 | 2/60 (3.3)  0/3 (0) | 0.906 | 1/60 (1.7)  0/3 (0) | 0.952 | 1/60 (1.7)  0/3 (0) | 0.952 | 8/60 (13.3)  0/3 (0) | 0.661 | 2/60 (3.3) ^+^  1/3 (33.3) ^+^ | 0.017* | 11/60 (18.3)  3/3 (100.0) | 0.009* | 1/60 (1.7)  0/3 (0) | 0.952 |

Acne, Acneiform rash grade 3; Bullous, Bullous dermatitis; Papule, Papulopustular rash; QTc, QTc prolongation; n, number of ADRs in the genotype; N, number of all cases in the genotype; (%), incidence rate

+, diarrhea grade 3

* Fisher's Exact Test statistic significant
